# Rack1 Mediates the Interaction of P-Glycoprotein with Anxa2 and Regulates Migration and Invasion of Multidrug-Resistant Breast Cancer Cells

**DOI:** 10.3390/ijms17101718

**Published:** 2016-10-13

**Authors:** Yi Yang, Na Wu, Zhiyong Wang, Fei Zhang, Ran Tian, Wei Ji, Xiubao Ren, Ruifang Niu

**Affiliations:** 1 Public Laboratory, Tianjin Medical University Cancer Institute and Hospital, National Clinical Research Center for Cancer, Tianjin 300060, China; yiyang@tmu.edu.cn (Y.Y.); wzy7848354@hotmail.com (Z.W.); feizhang03@tmu.edu.cn (F.Z.); tianrants1985@163.com (R.T.); jiwei217@126.com (W.J.); 2 Key Laboratory of Cancer Prevention and Therapy, Tianjin Medical University Cancer Institute and Hospital, Tianjin 300060, China; tjmuzhangfei@hotmail.com; 3 Tianjin’s Clinical Research Center for Cancer, Tianjin Medical University Cancer Institute and Hospital, Tianjin 300060, China; 4 Key Laboratory of Breast Cancer Prevention and Therapy, Tianjin Medical University, Ministry of Education, Tianjin 300060, China; 5 Department of Immunology, Tianjin Medical University Cancer Institute and Hospital, National Clinical Research Center for Cancer, Tianjin 300060, China; 6 Key Laboratory of Cancer Immunology and Biotherapy, Tianjin Medical University Cancer Institute and Hospital, Tianjin 300060, China

**Keywords:** P-glycoprotein, Rack1, Anxa2, MDR, invasion, breast cancer, Src

## Abstract

The emergence of multidrug resistance is always associated with more rapid tumor recurrence and metastasis. P-glycoprotein (P-gp), which is a well-known multidrug-efflux transporter, confers enhanced invasion ability in drug-resistant cells. Previous studies have shown that P-gp probably exerts its tumor-promoting function via protein-protein interaction. These interactions were implicated in the activation of intracellular signal transduction. We previously showed that P-gp binds to Anxa2 and promotes the invasiveness of multidrug-resistant (MDR) breast cancer cells through regulation of Anxa2 phosphorylation. However, the accurate mechanism remains unclear. In the present study, a co-immunoprecipitation coupled with liquid chromatography tandem mass spectrometry-based interactomic approach was performed to screen P-gp binding proteins. We identified Rack1 as a novel P-gp binding protein. Knockdown of Rack1 significantly inhibited proliferation and invasion of MDR cancer cells. Mechanistic studies demonstrated that Rack1 functioned as a scaffold protein that mediated the binding of P-gp to Anxa2 and Src. We showed that Rack1 regulated P-gp activity, which was necessary for adriamycin-induced P-gp-mediated phosphorylation of Anxa2 and Erk1/2. Overall, the findings in this study augment novel insights to the understanding of the mechanism employed by P-gp for promoting migration and invasion of MDR cancer cells.

## 1. Introduction

Chemotherapy is one of the major methods for treating late-stage malignant tumors [1,2]. However, the emergence of drug resistance, especially multidrug resistance, considerably hinders effective cancer treatment [1,2]. Development of multidrug resistance not only results in clinical failure, but is also associated with more rapid tumor recurrence and metastasis [3,4,5]. In vitro studies demonstrated that chronic exposure of cancer cells to chemotherapeutic drugs can lead to enhanced cell migration, and acquisition of drug resistance by tumor cells is usually accompanied by alterations in cell invasiveness [6,7,8]. Consequently, multidrug-resistant (MDR) cancer cells always exhibit enhanced metastatic potential compared to parental cells in vivo [8,9]. Enhanced invasive and metastatic ability is associated with upregulated expression of metastasis-related genes [10,11] and epithelial to mesenchymal transition-associated proteins in MDR cancer cells [6,8,12]. These phenomena suggest a functional link between drug resistance and cancer cell invasion and metastasis [5]. However, the detailed mechanism of this process remains unknown. Thus, overcoming cancer progression caused by chemotherapy failure is urgently needed for cancer treatment.

P-glycoprotein (P-gp) is a well-known multidrug-efflux transporter, and its overexpression in cancer cells is correlated with MDR phenotype [1]. P-gp induces multidrug resistance by acting as a pump that effluxes cytotoxic reagents out of the cell [1,2]. In addition to being a drug transporter, P-gp acts as a promoter during cancer progression [5]. Upregulated P-gp expression in cancer tissues is usually associated with a more malignant phenotype and poor clinical outcome for cancer patients [3,4,7,11,13,14]. Moreover, P-gp activity was associated with angiogenesis [15,16], cancer stem cells [5,17,18], cell migration and invasion [7,19,20,21,22], which are essential for carcinogenesis and cancer progression. These findings suggest that upregulated P-gp may confer enhanced cell invasion ability in drug-resistant cells. However, the underlying mechanisms employed by P-gp to promote cell invasiveness remain poorly understood.

In addition to its action as a membrane transporter, upregulation of P-gp expression has been correlated with activation of several cellular signaling, especially for the Erk1/2 pathway [23]. Interestingly, treatment of drug-resistant cells with P-gp substrates or with specific antibodies resulted in phosphorylation of Erk1/2 along with enhanced cell invasiveness [21,24]. On the contrary, inhibition of P-gp or knockdown of P-gp expression suppressed P-gp substrate-induced cellular activities [7,21]. In addition, protein-protein interactions have been implicated in the regulation of P-gp activity and cellular activities, such as migration and invasion of drug-resistant cells [7,20]. Protein–protein interaction is necessary for signal transduction [25,26]. These results indicated that P-gp exerts its tumor-promoting functions probably by mediating intracellular signal transduction. Nevertheless, the mechanism by which P-gp functions in cellular signaling is largely unknown. Thus, identifying the interactome of P-gp is pivotal and important for enhancing the understanding of P-gp-mediated molecular pathways responsible for cancer progression induced by drug resistance.

We previously reported that P-gp activity regulates Anxa2 phosphorylation and promotes the invasiveness of MDR breast cancer cells [7]. In the present study, the precise mechanism by which P-gp modulates Anxa2 phosphorylation was defined. A co-immunoprecipitation (co-ip) coupled with liquid chromatography tandem mass spectrometry (LC-MS/MS)-based interactomic approach was performed to screen P-gp binding proteins. We showed that Rack1 was a novel P-gp binding protein. Silencing of Rack1 expression significantly inhibited the proliferation and invasion of MDR breast cancer cells. Mechanistic studies demonstrated that Rack1 mediated the binding of P-gp to Anxa2 and Src. Moreover, Rack1 regulated P-gp activity and was necessary for adriamycin-induced P-gp-mediated tyrosine phosphorylation of Anxa2 and Erk1/2 phosphorylation. Thus, the results add novel insights to the understanding of the mechanism employed by P-gp for promoting the migration and invasion of MDR cancer cells.

## 2. Results

### 2.1. Identification of P-Glycoprotein Binding Proteins Using Interactome Analysis

**Figure 1:**
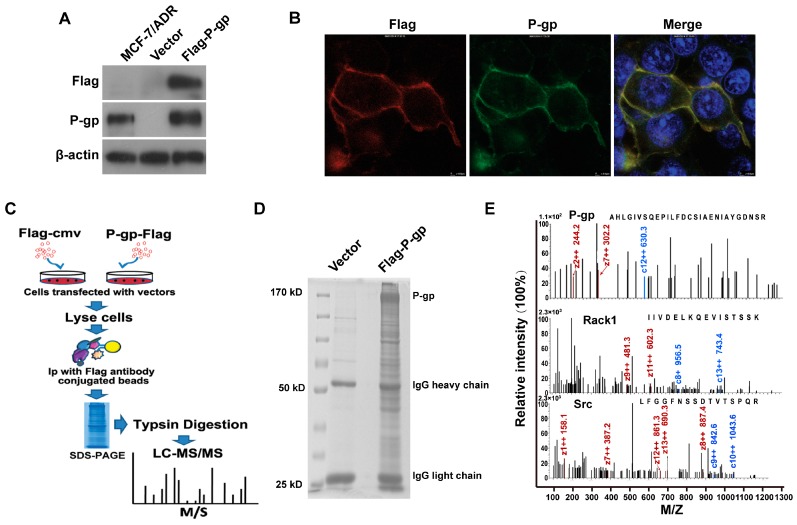
Identification of P-glycoprotein (P-gp) binding proteins using co-immunoprecipitation (co-ip)-coupled liquid chromatography tandem mass spectrometry (LC-MS/MS)-based interactome analysis. (**A**) Western blotting analysis of P-gp and Flag expression in HEK 293T cells transfected with Flag-tagged P-gp plasmid or empty vector. Cell lysates from P-gp expressing MCF-7/ADR (Adriamycin Resistance) cells were used as a positive control; (**B**) Confocal immunofluorescence microscopy analysis showed that P-gp was expressed normally in the cell membrane (Red: Flag, Green: P-gp; Yellow: Merge; 600× magnification); (**C**) Flowchart of sample preparation and mass spectrographic analysis in this study; (**D**) Sodium dodecyl sulfate polyacrylamide gel electrophoresis (SDS-PAGE) analysis of P-gp binding proteins. The P-gp binding proteins were separated by SDS-PAGE and stained with Coomassie brilliant blue R-250; (**E**) Representative mass spectrum of P-gp interacting proteins identified by MS/MS analysis.

We previously reported that P-gp interacts with Anxa2 and promotes the invasiveness of MDR breast cancer cells by regulating Anxa2 phosphorylation [7]. However, the detailed mechanism by which P-gp modulates Anxa2 phosphorylation remains unclear. Protein-protein interaction is the general route for cellular signal transduction. Thus, identifying the interactome of P-gp is necessary to elucidate the function of P-gp in promoting cancer cell invasion. In this study, a Flag-tagged P-gp-expressing vector was constructed and introduced into HEK 293T cells. As shown in Figure 1A, Western blotting analysis showed an apparent P-gp expression in HEK 293T cells as detected using anti-Flag or anti-P-gp antibodies. Immunofluorescence analysis also confirmed that P-gp was expressed normally in the cell membrane (Figure 1B). Then, the protein interaction network of P-gp was investigated using a co-immunoprecipitation combined with LC-MS/MS-based approach. The workflow in this study is summarized in Figure 1C. The enriched proteins interacting with P-gp are shown in Figure 1D. A total of 515 proteins were identified as candidate P-gp binding proteins (Supplementary Table S1). Some representative MS/MS spectra of peptides are presented in Figure 1E.

### 2.2. Rack1 Is a Novel Binding Protein of P-Glycoprotein

**Figure 2:**
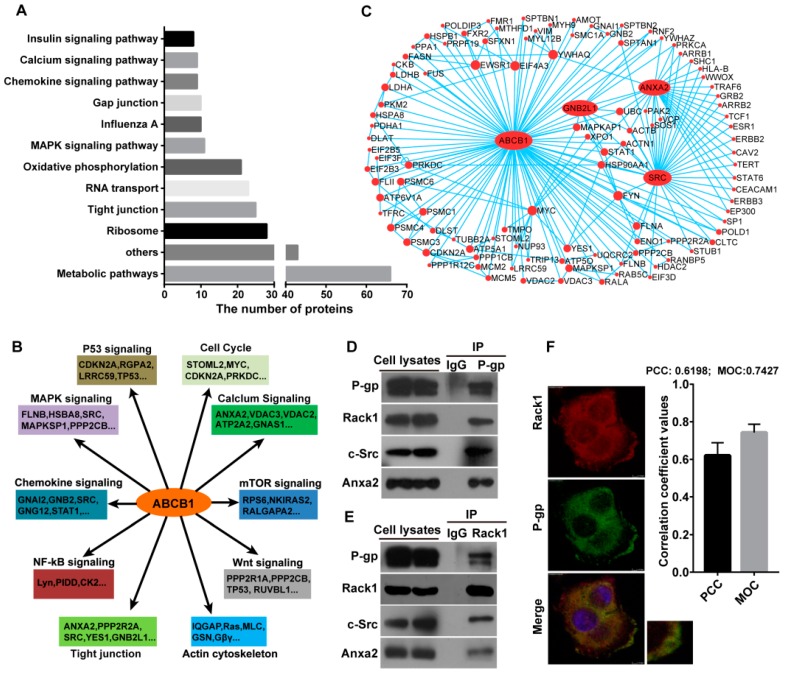
Functional characterization and validation of candidate P-gp interacting proteins identified that Rack1 was a novel binding protein of P-gp. (**A**) Identified P-gp interacting proteins were categorized by signal pathway in Kyoto Encyclopedia of Genes and Genomes (KEGG) database; (**B**) The association of the major P-gp binding proteins with cellular signaling pathways involved in cancer; (**C**) Network of P-gp interacting proteins. A total of 108 P-gp interacting proteins were mapped via protein-protein interaction databases searches using the VisAnt software and visualization in Cytoscape; only proteins that interact with at least two other proteins are shown. Nodes represent proteins; lines represent interactions; and node size indicates the interaction degree; (**D**) Co-immunoprecipitation assay showed that endogenous P-gp interacted with endogenous Rack1, c-Src and Anxa2 in drug-resistant breast cancer cells. MCF-7/ADR cells were lysed, immunoprecipitated with anti-P-gp antibody and then analyzed by western blotting with anti-P-gp, Rack1, c-Src or Anxa2 antibody; (**E**) Endogenous Rack1 interacted with endogenous P-gp, c-Src and Anxa2 in breast cancer cells. MCF-7/ADR cells were lysed, immunoprecipitated with anti-Rack1 antibody, followed by Western blotting analysis; (**F**) Confocal immunofluorescence microscopy analysis showed that Rack1 was colocalized with P-gp in MCF-7/ADR cells (Red: Rack1, Green: P-gp, Yellow: Merge; 600× magnification). Image J software (NIH, Bethesda, MD, USA) was used to quantify the colocalization efficiency. The plasma membrane regions of the cells were selected as the region of interest. Both Pearson’s correlation coefficient (PCC) and Mander’s overlap coefficient (MOC) were used to calculate the degree of colocalization between Rack1 and P-gp. Thirty images were analyzed.

Kyoto Encyclopedia of Genes and Genomes (KEGG) pathway analysis of P-gp binding proteins was performed using the Database for Annotation, Visualization and Integrated Discovery (DAVID) online analysis tool [27]. Several of the associated signaling pathways with the highest protein hits are summarized in Figure 2A,B. The P-gp binding proteins were associated with multiple signal pathways, including metabolic, tight junction, Mitogen-activated protein kinases (MAPK) signaling and chemokine signaling. These findings are consistent with those in previous studies, in which P-gp functions in the excretion of toxic metabolites [2,5], G protein–coupled receptor (GPCR) signaling [5], tight junction integrity [28,29], Erk1/2 activation [21,23,24] and calcium signaling [30,31]. Moreover, a connected network comprising 108 P-gp binding proteins was constructed using protein–protein interaction database searches and visualized using the Cytoscape software (Figure 2C). In addition to P-gp (ABCB1), Anxa2 (ANXA2), Rack1 (GNB2L1) and Src (SRC) displayed more interactions than other proteins in this network, indicating that these proteins may function as “hubs” in the P-gp interaction protein network (Figure 2C). We validated our hypothesis by performing a co-immunoprecipitation assay in drug-resistant breast cancer cells MCF-7/ADR using anti-P-gp antibodies followed by Western blot analysis. As shown in Figure 2D, P-gp co-precipitated with Anxa2, Src and Rack1 in an endogenous condition. In addition, the reciprocal co-immunoprecipitation assay using anti-Rack1 antibodies confirmed that Rack1 was a novel binding protein of P-gp (Figure 2E). Immunofluorescence confocal microscopy analysis results also showed that Rack1 was colocalized with P-gp in the membrane (Figure 2F), supporting the existence of an interaction between P-gp and Rack1. We previously showed that Rack1 was also a binding protein of Anxa2 [32] and that Rack1 was a known substrate of tyrosine protein kinase Src [33]. Thus, these results suggested that these four proteins may form a protein complex in physiological conditions.

### 2.3. Knockdown of Rack1 Inhibits Proliferation, Migration and Invasiveness of Multidrug-Resistant Breast Cancer Cells

**Figure 3:**
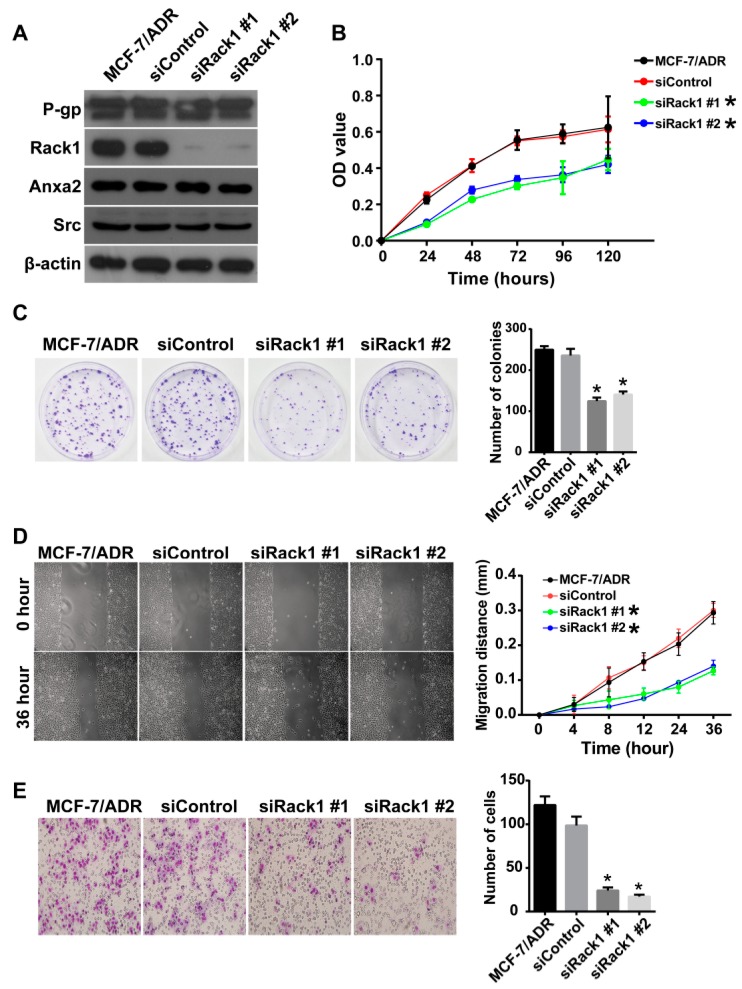
Knockdown of Rack1 inhibits the proliferation, migration and invasiveness of multidrug-resistant breast cancer cells. (**A**) Western blotting analysis of P-gp, Rack1, c-Src and Anxa2 expression in MCF-7/ADR cells transfected with control or Rack1 specific stealth siRNAs (# represents siRNA sequence ); (**B**) Silencing of Rack1 expression inhibited cell proliferation compared with that of control cells. Wild type, control and Rack1 knockdown cells were seeded in 96-well plates at a density of 4 × 10^3^ cells per well and consecutively cultured for 24, 48, 72, 96 and 120 h. The cell proliferative activity was calculated using the CCK-8-based method by measuring the absorbance at 450 nm on a micro-ELISA reader. The assays were carried out using six replicates for each time point and repeated three times; (**C**) Knockdown of Rack1 induced a significant decrease in the colony formation ability of breast cancer cells. Control and Rack1 knockdown cells were seeded in 35-mm dishes at a density of 500 cells per dish and cultured for 10 days. The number of colonies was quantified under an inverted microscope; (**D**) Knockdown of Rack1 significantly inhibited cell migration as measured by the wound healing assay (100× magnification). Relative cell migration distance was quantified and plotted in the right panel. Data as the mean ± SD of triplicates, *p* < 0.0001; (**E**) Knockdown of Rack1 expression inhibited the invasion ability of breast cancer cells (200× magnification). Approximately 200 μL of cell suspension (approximately 8 × 10^4^ cells) in serum-free medium were added to the upper transwell inserts pre-coated with Matrigel; the lower insert was loaded with 10% FBS-containing cell culture medium. After 36 h of incubation at 37 °C, invaded cells were fixed, stained and counted under an inverted microscope. The average result of triplicate experiments is summarized in the right panel. Data as the mean ± SD, *p* < 0.0001 versus control cells. Statistical analysis was performed by one-way ANOVA (* *p* < 0.05).

Abnormal expression of Rack1 was observed in many types of cancer [34,35]. However, the detailed function of Rack1 in cancer cell proliferation and invasiveness remains undefined because of conflicting reports [34,36,37]. In this study, Rack1 expression was downregulated using siRNA to determine the role of Rack1 in the proliferation and invasion of MDR cells. As shown in Figure 3A, Rack1 was significantly silenced in MCF-7/ADR cells transfected with two different Rack1 specific siRNAs. CCK-8 and the colony formation assay demonstrated that depletion of Rack1 remarkably inhibited cell proliferation compared with that of control cells (Figure 3B,C). In addition, the wound healing assay showed that knockdown of Rack1 resulted in an apparent decrease in cell migration (Figure 3D). Cell invasion ability was also significantly inhibited in Rack1 depletion cells as measured by the Transwell assay (Figure 3E). These results are consistent with previous reports that Rack1 was a positive regulator in breast cancer proliferation and invasion [37,38]. Thus, our study supports the tumor promoter role of Rack1 in breast cancer.

### 2.4. Knockdown of Rack1 Attenuates the Interaction of P-Glycoprotein with Anxa2 and Src

**Figure 4:**
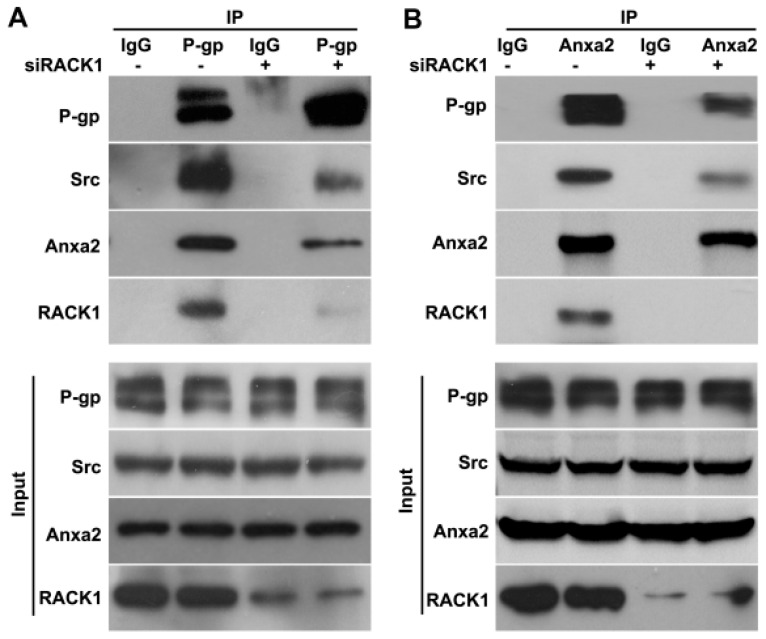
Knockdown of Rack1 attenuates the interaction of P-glycoprotein with Anxa2 and Src. (**A**) The interaction of P-gp with Anxa2 and Src in Rack1 depletion cells was significantly attenuated compared with that of control cells. Control and Rack1 knockdown MCF-7/ADR cells were lysed, immunoprecipitated with anti-P-gp antibody and then analyzed by Western blotting with anti-P-gp, Rack1, c-Src or Anxa2 antibody; (**B**) Silencing of Rack1 expression significantly attenuated the interaction of Anxa2 with P-gp and Src. Control and Rack1 knockdown MCF-7/ADR cells were lysed, immunoprecipitated with anti-Anxa2 antibody and then analyzed by Western blotting with anti-P-gp, Rack1, c-Src or Anxa2 antibody.

Rack1 was an important scaffold protein in mediating protein–protein interaction and cellular signal transduction [39,40]. We hypothesized that Rack1 may mediate the interaction between P-gp and Src/Anxa2. To investigate whether Rack1 expression is necessary for the formation of the P-gp-Anxa2-Src complex, the co-immunoprecipitation assay was performed in Rack1 knockdown MCF-7/ADR cells using anti-P-gp antibodies followed by Western blotting analysis. As shown in Figure 4A, the interaction between P-gp and Src or Anxa2 in Rack1 depletion cells was significantly attenuated compared with that of control cells. In addition, a reciprocal co-immunoprecipitation experiment was performed using anti-Anxa2 antibodies in cell lysates from Rack1 depletion cells. The binding of P-gp and Src to Anxa2 was obviously reduced in Rack1 silenced cells, further confirming that Rack1 is required for the formation of P-gp-Src-Anxa2 protein complex (Figure 4B). Thus, these results suggested that Rack1 may function as the molecular bridge linking P-gp to the recruitment of Src and Anxa2.

### 2.5. Knockdown of Rack1 Inhibits Anxa2 Phosphorylation and Adriamycin-Induced Erk1/2 Phosphorylation

**Figure 5:**
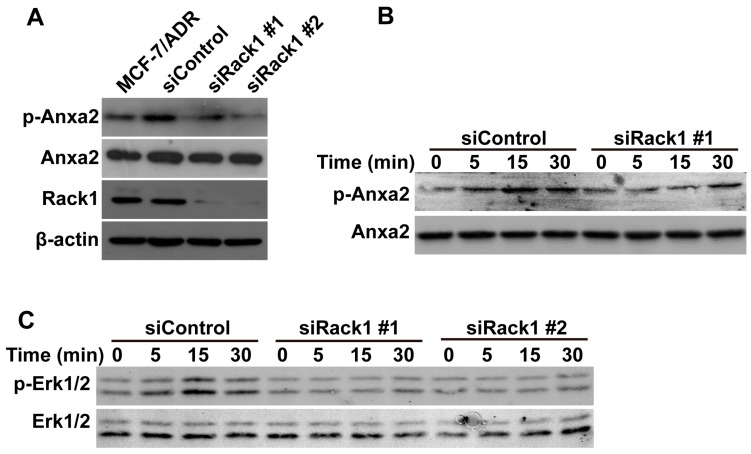
Knockdown of Rack1 inhibits Anxa2 phosphorylation and adriamycin induced Erk1/2 phosphorylation. (**A**) Western blotting analysis of Anxa2 and tyrosine phosphorylated Anxa2 expression in MCF-7/ADR cells transfected with control or Rack1 specific stealth siRNAs; (**B**) Silencing of Rack1 inhibited adriamycin induced phosphorylation of Anxa2 in MCF-7/ADR cells. Control and Rack1 knockdown cells were starved for 24 h and then exposed with 0.5 µM adriamycin for different time points, then cells were lysed, and total cell lysate was further analyzed by Western blotting; (**C**) Downregulation of Rack1 inhibited adriamycin-induced phosphorylation of Erk1/2 in MCF-7/ADR cells. Western blotting analysis of adriamycin-induced phosphorylation of Erk1/2 in MCF-7/ADR cells transfected with control and Rack1-specific stealth siRNAs.

Tyrosine phosphorylation of Anxa2 is critical for cancer cell migration and invasion [41]. P-gp was previously reported to interact with Anxa2 and to regulate its tyrosine phosphorylation [7]. We performed the Western blot assay to investigate the possible effect of the attenuated interaction between P-gp and Anxa2 induced by Rack1 reduction on Anxa2 phosphorylation. As shown in Figure 5A, silencing of Rack1 inhibited the basal level of Anxa2 phosphorylation. Consistently, knockdown of Rack1 also inhibited adriamycin-induced phosphorylation of Anxa2 (Figure 5B). Moreover, knockdown of Rack1 also inhibited adriamycin-induced Erk1/2 phosphorylation (Figure 5C), which agrees with the previous report that Anxa2 promotes the migration and invasion of MCF-7 cells through regulation of Erk1/2 signaling. Therefore, these data suggest that Rack1 was necessary for adriamycin-induced Anxa2 phosphorylation.

### 2.6. Knockdown of Rack1 Inhibited P-gp Activity, and P-gp Is Required for Adriamycin-Induced Erk1/2 Phosphorylation

**Figure 6:**
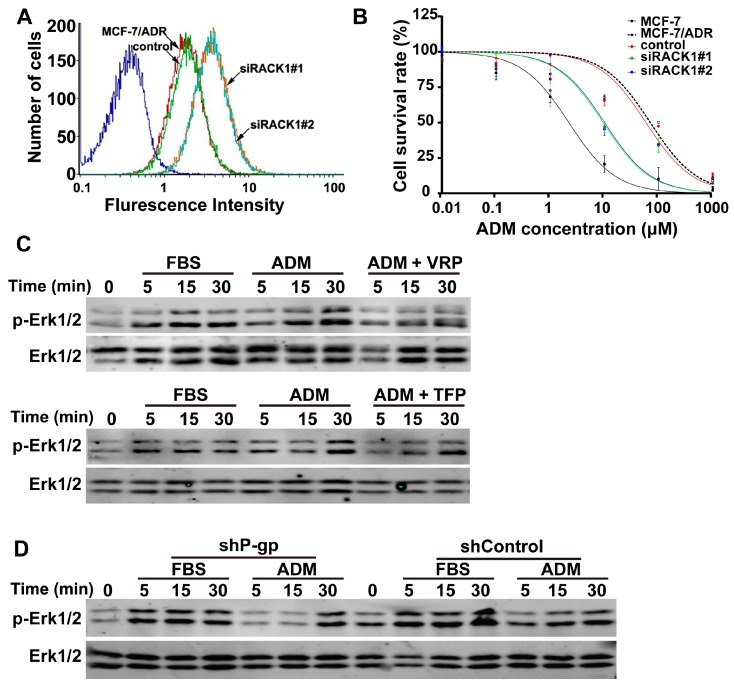
Knockdown of Rack1 inhibited P-glycoprotein activity and P-gp is required for adriamycin (ADM)-induced Erk1/2 phosphorylation. (**A**) Knockdown of Rack1 significantly reduced the efflux rate of fluorescent dye Rh-123 in MCF-7/ADR cells as measured by using flow cytometry; (**B**) Knockdown of Rack1 enhanced the drug sensitivity to adriamycin in MCF-7/ADR cells. Control and Rack1 silenced cells were seeded in a 96-well plate at a density of 1 × 10^4^ cells per well and cultured for 24 h. Then, different concentrations of adriamycin were added into each well. After incubation for 72 h at 37 °C, the cell viability was determined using the CCK-8-based method by measuring the absorbance at 450 nm on a micro-ELISA reader. The assays were performed using three replicates for each drug concentration. IC_50_ was calculated by using the GraphPad Prism 6.00 software; (**C**) Inhibition of P-gp activity inhibited adriamycin-induced Erk1/2 phosphorylation. Cells were starved for 24 h in the presence or absence of TFP or VRP and then exposed with different concentrations of adriamycin for different time points, then cells were lysed, and total cell lysate was further analyzed by Western blotting. FBS was used as positive control; (**D**) Knockdown of P-gp impaired adriamycin-induced phosphorylation of Erk1/2 in MCF-7/ADR cells. Control and P-gp knockdown cells were starved for 24 h and then exposed with different concentration of adriamycin for different time points; the cells were lysed and total cell lysate was further analyzed by Western blotting. FBS was used as a positive control. VRP, verapamil; TFP, trifluoperazine.

P-gp activity regulates Anxa2 and Erk1/2 phosphorylation in drug-resistant cells [7,21]. Adriamycin is a substrate of P-gp. Thus, the decrease in Anxa2 and Erk1/2 phosphorylation in Rack1 silenced cells may be ascribed to the attenuation of P-gp activity. We investigated this possibility by performing the Rh123 efflux assay using flow cytometry. As shown in Figure 6A, the retention of fluorescent dye Rh123 in Rack1 knockdown cells was significantly increased compared with that of control cells, indicating the inhibition of P-gp activity. Consequently, the drug sensitivity of Rack1 depletion cells was remarkably increased compared with that of control cells as measured by the IC_50_ assay (Figure 6B). Next, two well-known P-gp inhibitors, verapamil (VRP) and trifluoperazine (TFP), were used to further explore the effect of P-gp inhibition on Erk1/2 phosphorylation. As shown in Figure 6C, drug-induced phosphorylation of Erk1/2 was obviously abrogated in VRP- or TFP-treated cells. These data suggested that P-gp activity was necessary for adriamycin-induced Erk1/2 phosphorylation. In addition, we investigated the effect of P-gp knockdown on Erk1/2 phosphorylation. In agreement with the above observations, knockdown of P-gp also significantly suppressed adriamycin-induced Erk1/2 phosphorylation compared with that of control cells. However, silencing of P-gp has no significant effect on serum-induced phosphorylation of Erk1/2 (Figure 6D). Therefore, these results suggested that Rack1 depletion resulted in a decrease in Erk1/2 phosphorylation because of the inhibition of P-gp activity.

## 3. Discussion

Development of multidrug resistance by cancer cells is always associated with enhanced cell invasiveness in vitro and increased metastatic potential in vivo [5,9,12,19,21,22]. The high expression of P-gp in many types of cancer tissues was reported to be correlated with a more aggressive phenotype [3,4,7,11,13,14]. In addition to being a drug transporter, in vitro studies also demonstrated additional functions of P-gp, such as for apoptosis and proliferation [5], cancer stem cells [5,17,18], angiogenesis [15,16], cell invasion and metastasis [19,20,21,22], which are critical for cancer initiation and progression. Previous studies have suggested that P-gp exerts its tumor-promoting function through protein–protein interaction [7,20,23]. Importantly, these interactions have been implicated in the activation of intracellular signal transduction, especially for the Erk1/2 pathway [21,23]. We previously showed that P-gp binds to Anxa2 and promotes the invasiveness of MDR breast cancer cells through regulation of Anxa2 phosphorylation [7]. However, the actual mechanism by which P-gp modulates Anxa2 phosphorylation remains unclear. In this study, we demonstrated that Rack1 acted as a novel P-gp binding partner and mediated the interaction between P-gp and Anxa2/Src. Knockdown of Rack1 significantly inhibited the proliferation and invasion of MDR breast cancer cells. We demonstrated that Rack1 was necessary for adriamycin-induced, P-gp-mediated tyrosine phosphorylation of Anxa2, as well as Erk1/2 phosphorylation. We further showed that Rack1 regulated P-gp activity and was responsible for adriamycin-induced Erk1/2 phosphorylation. Hence, the data presented in this study elucidated the mechanism employed by P-gp to promote the migration and invasion of MDR cancer cells. Our findings also provided new insights on the understanding of the mechanism underlying the functional association between MDR and cancer invasiveness.

Conventionally, P-gp was considered physiologically as a drug transporter that effluxes cytotoxic agents from cells [2,5]. Nevertheless, several studies have shown that P-gp substrates, such as adriamycin, induce the upregulation of metalloproteinase-2/9 in drug-resistant cells, and this phenomenon can be blocked using P-gp neutralizing antibodies [10,24]. Adriamycin also induces phosphorylation of Anxa2, and this effect can be inhibited by P-gp inhibitors or knockdown of P-gp expression [7]. In addition, upregulation of P-gp expression in wild-type cancer cells resulted in constitutive activation of Erk1/2 signaling [23]. Similarly, P-gp stimulation also increases Erk1/2 phosphorylation in drug-resistant cells [21]. These findings indicated that P-gp may also be a mediator in cellular signal transduction. In the present study, using the KEGG database analysis, we showed that the enriched P-gp binding proteins were also implicated in multiple signal pathways (Figure 2A). Several of P-gp’s functions have been reported previously, such as MAPK signaling [21,23,24], tight junction [28,29] and calcium signaling [30,31]. Thus, the results from the present study further demonstrated that P-gp may also function in transducing intracellular signaling. However, the manner by which P-gp exerts these functions remains unclear. In this study, we showed that Anxa2, Rack1 and Src may function as “hubs” in the P-gp interaction protein network. These interactions were further confirmed using the endogenous co-immunoprecipitation assay. Hence, our findings also provided direct clues to uncover the molecular basis underlying P-gp as a signal mediator. To our knowledge, this study is the first report on P-gp interactome analysis.

Rack1 is a multifunctional scaffold protein that can function as a platform for clustering signaling proteins to facilitate cellular signal transduction [34,39,40]. Rack1 is a known substrate of protein-tyrosine kinase Src [33], which is also a main upstream kinase of Anxa2 [42]. The high expression of Anxa2, which is a critical signal molecule, is important for the migration and invasion of MDR cells [43,44]. Anxa2 and Src are also binding partners of P-gp [7]. Thus, we hypothesized that Rack1 may mediate the formation of these protein complex. Our results showed that Rack1 interacted with P-gp, Anxa2 and Src in the endogenous condition in drug-resistant cells. Moreover, we showed that silencing of Rack1 expression not only decreased the interaction between P-gp and Anxa2/Src, but also attenuated the binding ability of Anxa2 to Src. These results indicated that Rack1 was necessary for maintaining the integrity of this protein complex. The protein–protein interaction was proven to be critical for signal transduction [25,26]. Therefore, reduced interaction between P-gp/Anxa2 and Src in Rack1 depletion cells may probably lead to the inhibition of phosphorylation of Anxa2 by Src. Accordingly, our findings showed that the basal level and adriamycin-induced phosphorylation of Anxa2 were significantly decreased in Rack1-silenced cells. Thus, our results suggest that Rack1 nucleated a signal complex among P-gp, Anxa2 and Src and enhanced necessary binding of Anxa2 to Src or P-gp, thereby facilitating the phosphorylation of Anxa2 by Src kinase.

Aberrant expression of Rack1 is observed in many types of carcinoma [34,37,45,46,47]. Several studies demonstrated that Rack1 was a key enhancer in cancer cell proliferation and invasion [37,38,48,49,50]. By contrast, other studies have shown that Rack1 functions as a suppressor in growth, invasion and metastasis in several types of cancer [36,46,51]. These conflicting reports suggest that the biological effect of Rack1 on cancer progression may be cell-type and context specific. Given that Rack1 is a well-known multifunctional scaffolding protein, which functions at the central position of signal transduction network and determines the specificity and efficiency of signaling events [34,39,40], it is possible that the dual faces of Rack1 in tumorigenesis may be attributed to the formation of diverse complexes with different signaling molecules. In other words, the functional diversity of Rack1 in cancer might depend on the properties of its binding proteins. In the present study, we showed that Rack1 makes a complex with Anxa2 and Src. Anxa2 is proven to be a tumor promoter in breast cancer progression. The non-receptor tyrosine kinase Src is a well-known proto-oncogene and is critical for cancer cell survival, proliferation, invasion and metastasis [52,53,54]. Thus, we supposed that Rack1 functions as a positive regulator in breast cancer. Consistent with this hypothesis, we demonstrated that silencing of Rack1 expression significantly inhibited the proliferation and invasiveness in MDR breast cancer cells, which is consistent with previous reports that Rack1 was a promoter in breast cancer progression [37,38]. Additionally, a recent study has shown that Rack1 is involved in drug resistance in hepatocellular carcinoma [55]. Consistent to this result, we also found that Rack1 was critical for resistance to chemotherapeutic drugs in MDR cells. Interestingly, knockdown of Rack1 expression did not affect the protein level of P-gp. The increased drug sensitivity of Rack1 depletion cells was due to the reduced P-gp activity. It is worth noting that Src also plays a very important role in drug resistance. Previous studies have shown that inhibition of Src kinase can sensitize drug-resistant cancer cells to chemotherapeutic agents without an apparent effect on P-gp expression [56]. Therefore, the interaction of Rack1 with Src may probably contribute to its regulatory effect on drug resistance. However, information on the effect of Rack1 on drug resistance is limited. The detailed mechanism by which Rack1 contributes drug resistance requires further exploration in the future. Altogether, our data support a tumor promoter role of Rack1 in breast cancer.

Previous studies have found a functional link between P-gp activity and activation of the Erk1/2 pathway [21,23]. Consistent with these observations, our findings also showed that inhibition of P-gp activity or knockdown of P-gp expression significantly suppressed adriamycin-induced phosphorylation of Erk1/2. These phenomena raised an interesting question on the mechanisms of P-gp-mediated activation of Erk1/2 signaling. Rack1 regulated P-gp activity, and Rack1 was implicated in the activation of Erk1/2 signaling [57,58]. We inferred that Rack1 may be involved in P-gp-mediated activation of Erk1/2. As supposed, knockdown of Rack1 significantly inhibited adriamycin-induced Erk1/2 phosphorylation in drug-resistant cells. Thus, our findings suggested that Rack1 was required for P-gp-mediated, adriamycin-induced phosphorylation of Erk1/2. Moreover, depletion of P-gp had no effect on serum-induced phosphorylation of Erk1/2, suggesting that P-gp expression was not necessary for growth factor-induced activation of Erk1/2. However, P-gp-mediated activation of cellular signaling probably provides another survival approach in drug-resistant cells and leads to an increase in cell invasiveness.

## 4. Materials and Methods

### 4.1. Cell Lines, Reagents and Antibodies

HEK-293T cells were obtained from American Type Culture Collection. Cells were cultured in DMEM high-glucose medium (Hyclone, Logan, UT, USA) containing 10% fetal bovine serum (FBS, Gibco BRL, Rockville, MD, USA) at 37 °C with 5% CO_2_. Human breast cancer MCF-7 cells and their MDR variants MCF-7/ADR cells were provided by Zizheng Hou of Henry Ford Hospital, Detroit, MI, USA. Control and P-gp stable knockdown MCF-7/ADR cells were obtained in our previous study [7]. The cells were cultured in RPMI-1640 medium supplemented with 10% FBS at 37 °C with 5% CO_2_. MCF-7/ADR cells were cultured in the presence of 0.5 μM adriamycin to sustain the drug-resistant phenotype. Control and P-gp stable knockdown cells were cultured in the presence of 150 μg/mL hygromycin B (BD Biosciences, San Jose, CA, USA). Adriamycin was obtained from Hisunpharm Co. (Taizhou, China). Lipofecatmine 2000 was obtained from Invitrogen (Carlsbad, CA, USA). The BCA protein assay kit was obtained from Thermo Fisher Scientific (Waltham, MA, USA). The Cell Counting Kit-8 (CCK-8) was obtained from Dojindo Laboratories (Kamimashiki-gun, Kumamoto, Japan). Transwell inserts and enhanced chemiluminescence reagents were obtained from Millipore (Darmstadt, Hesse, Germany). Protease inhibitor (EDTA-free) was purchased from Roche Diagnostics (Mannheim, Germany). Rabbit polyclonal antibodies against P-gp and Src, as well as mouse monoclonal antibodies against Anxa2, p-Anxa2, Rack1 and β-actin were obtained from Santa Cruz Inc. (Santa Cruz, CA, USA). Rabbit monoclonal antibodies against Erk1/2 and p-Erk1/2 (Thr202/Tyr204) were obtained from Cell Signaling Technology (Danvers, MA, USA). Alexa Fluor 488-conjugated goat anti-rabbit and Alexa Fluor 568-conjugated goat anti-mouse secondary antibodies were obtained from Invitrogen (Carlsbad, CA, USA). Rhodamine 123 (Rh123) dye, antibody against Flag and anti-Flag antibody-conjugated agarose beads were obtained from Sigma (St. Louis, MO, USA). Protein A and G agarose beads were obtained from Invitrogen (Carlsbad, CA, USA).

### 4.2. Vector Construction, siRNA and Plasmid Transfection

Full-length MDR1 was amplified by PCR from the pHaMDRwt plasmid, which was a gift from Michael Gottesman (Addgene Plasmid # 10957), and then subcloned into a pFLAG-neo-C expression vector in the BamH I and Xho I sites. The plasmid was further confirmed by double enzyme digestion and DNA sequencing. Control and two Rack1-specific stealth siRNAs (Sequence #1: 5′-UAUCUCGAGAUCCAGAGACAAUCUG-3′; and sequence #2: 5′-ACGAUGAUAGGGUUGCUGCUGUUGG-3′) were obtained from Invitrogen (Carlsbad, CA, USA). Transient transfections were performed as described previously [32]. In brief, cells were seeded in a six-well plate and cultured to 70%–80% (for plasmid transfection) or 30%–40% (for siRNAs) confluence. Then, 4 μg of endotoxin-free plasmid or 5 μL of stealth siRNA were transfected into cells using Lipofectamine 2000 according to the manufacturer’s instructions. After transfection for 48 h, the cells were harvested and used for further experiments.

### 4.3. Western Blotting Analysis

Western blotting analysis was performed as described previously [7]. In brief, total cellular proteins were extracted using 1× SDS sample buffer and quantified by the BCA method. Approximately 20 μg of were separated by SDS-PAGE and transferred to a PVDF membrane. The membrane was blocked with 5% milk in 1× TBST and then incubated with primary antibodies at 4 °C overnight. Dilutions of the antibodies were as follows: P-gp, 1:500; Anxa2, 1:5000; p-Anxa2, 1:200; Rack1, 1:2000; Flag, 1:5000; Src, 1:500; Erk1/2, 1:2000; p-Erk1/2, 1:2500; and β-actin, 1:5000. The membrane was washed with 1× TBST and incubated with horseradish peroxidase-conjugated antibodies. Expression levels of targeted proteins were subsequently detected using an ECL kit according to the manufacturer’s protocol.

### 4.4. Co-Immunoprecipitation Assay

The P-gp binding proteins were enriched by performing a co-immunoprecipitation assay as described previously [32]. In brief, HEK-293T cells were seeded in 6-cm dishes and cultured to 70%–80% confluence. The cells were transfected with control and P-gp-Flag plasmid using the Lipofectamine 2000 method. After transfection overnight, the cells were passaged into 10-cm dishes and further cultured for another 48 h. The cells were washed with PBS and lysed in 800 μL of Triton X100-based cell lysis buffer. After centrifugation of cell lysates at 12,000× *g* for 30 min at 4 °C, the supernatant was precleared with Protein G-linked agarose beads. The P-gp interacting proteins were enriched by co-immunoprecipitation using anti-Flag antibody-conjugated beads. The beads were washed with cell lysis buffer and boiled with 1× SDS sample buffer at 95 °C for 5 min. The immunoprecipitated proteins were separated by SDS-PAGE and stained with Coomassie brilliant blue R-250. MCF-7/ADR cells were lysed in Triton X100-based cell lysis buffer to analyze the protein–protein interactions. The protein lysates were initially immunoprecipitated using anti-P-gp or Rack1 or Anxa2 antibodies. The immunocomplex was further analyzed by Western blot using corresponding antibodies as described in Section 2.3. Normal rabbit/mouse IgG was used as negative controls.

### 4.5. In-Gel Trypsin Digestion, Protein Identification by LC-MS/MS and Data Analysis

Mass spectrometric analysis was performed as described previously [32]. In brief, protein bands stained with Coomassie brilliant blue were excised from the gels, cut into small pieces and dehydrated with 100% acetonitrile (ACN). The samples were reduced with 10 mM DTT for 1 h and alkylated with 55 mM iodoacetamide for 1 h in the dark. Afterward, the samples were dehydrated with 100% ACN, dried and digested with trypsin at 37 °C overnight. After tryptic digestion, the peptide fragments were extracted with 0.1% trifluoroacetic acid (TFA) in 50% ACN, dried and re-solubilized in Buffer A (2% ACN, 0.1% FA). Approximately 5.0 μg of the resulting peptides were separated using a nano-UPLC system (LC-20AD, Shimadzu Co., Kyoto, Japan). The samples were loaded into a trap column (2 cm × 100 μm, inside diameter (i.d.)) at 5 μL/min for 4 min before being put online with the analytical C18 reversed phase column (15 cm × 75 μm i.d.). The peptides were then eluted using a linear gradient of 2%–35% Solvent B (98% ACN, 0.1% FA) at 400 nL/min for 33 min; 35%–80% Solvent B for 2 min; 80% Solvent B for 4 min; and Solvent A for 1 min. The eluted peptides were analyzed using a Triple TOF 5600 mass spectrometer (AB SCIEX, Concord, ON, Canada) with a Nanospray III source (AB SCIEX, Concord, ON, Canada). A spray voltage of 2.5 kV was used. The curtain gas was set to 30 psi; the ion source gas was 15 psi; and the interface heater temperature was 150 °C. High-resolution mass spectral data were acquired from 100–1800 *m*/*z* using a 250-ms accumulation time per spectrum. The top 30 abundant ions were selected for collision-activated dissociation. Datasets were subsequently searched against the UniProt Human database using the Mascot software (Matrix Science, London, UK) with the following parameters: one missed tryptic cleavage of trypsin digestion, carbamidomethyl (C) as a static modification, asparagine deamidation as variable modification, oxidation of methionine, a peptide mass tolerance of 0.05 Da and an MS/MS tolerance of 0.1 Da. Only scores higher than the false discovery rate threshold (*p* < 0.05) were considered correctly identified. For protein characterization, the identified proteins of P-gp were used for analysis of KEGG pathways using the DAVID Bioinformatics Resources [27]. For network construction, any known proteins that interacted with P-gp were obtained through literature retrieval and database search using VisAnt software [59]. The interaction information and the interactome analysis data obtained in this study were then integrated, formatted and visualized using Cytoscape software (only proteins that interact with at least two other proteins were chosen).

### 4.6. Immunofluorescence Confocal Microscopy Analysis

Cells were plated on sterile glass coverslips in 12-well plates at a density of 5 × 10^4^ cells/mL per well. The cells were washed thrice with PBS at room temperature and then fixed with freshly prepared 4% PFA in PBS for 10 min. After repeated washing with PBS, the cells were permeabilized in 0.1% Triton X100 in PBS for 10 min and blocked in 3% BSA at room temperature for 1 h. The cells were stained with primary antibodies against P-gp (1:100) and Rack1 (1:200) at 4 °C overnight. Afterward, the cells were probed with Alexa Fluor 488- and Alexa Fluor 568-conjugated secondary antibodies at room temperature for 1 h, and the nuclei were stained with DAPI. Finally, the coverslips were mounted with Mowiol-based anti-fading reagents and visualized using laser scanning confocal microscopy (Leica TCS SP5, Leica Microsystems, Wetzlar, Hesse-Darmstadt, Germany). Colocalization efficiency was quantified through Image J software (NIH, Bethesda, MD, USA). The plasma membrane regions of the cells were selected as the region of interest. Both Pearson’s correlation coefficient (PCC) and Mander’s overlap coefficient (MOC) were used to calculate the degree of colocalization between Rack1 and P-gp.

### 4.7. Cell Viability Assay

The Cell Counting Kit-8 (CCK-8) was used to evaluate cell proliferation ability. Control and Rack1 knockdown cells were seeded in a 96-well plate at a density of 4 × 10^3^ cells per well and cultured for 24, 48, 72, 96 and 120 h. After each day, the culture medium was discarded, and 10 μL of CCK-8 reagent in 150 μL of medium were added into each well. The cells were incubated for 3 h at 37 °C. Then, cell proliferative activity was calculated by measuring the absorbance at 450 nm on a micro-ELISA reader. Culture medium without CCK-8 reagent was used as the negative control. The assays were conducted using six replicates for each time point and were repeated three times.

### 4.8. Colony Formation Assay

The colony formation assay was performed as described previously [32]. In brief, control and Rack1 knockdown cells were seeded in 3.5-cm dishes at a density of 0.5 × 10^3^ cells per dish and then consecutively cultured for 10 days. Afterward, the cells were washed thrice with PBS at room temperature, fixed with methanol and stained with the Three-Step Stain Set kit according to the manufacturer’s protocol. The number of colonies (≥50 individual cells) was counted using a light inverted microscope. The assay was performed in triplicate.

### 4.9. IC_50_ Assay

IC_50_ assay was evaluated using the CCK-8 according to the manufacturer’s protocol. Control and Rack1 silenced cells were seeded in a 96-well plate at a density of 1 × 10^4^ cells per well and cultured for 24 h. Then, the culture medium was discarded, and different concentrations of adriamycin in 200 μL of fresh medium were added into each well. After incubation for 72 h at 37 °C, the medium was removed, and 10 μL of CCK-8 reagent in 150 μL of fresh medium were added into each well. The cells were further incubated for 3 h, and cell viability was determined at 450 nm on a micro-ELISA reader. The assays were performed using three replicates for each drug concentration. The IC_50_ was calculated using the GraphPad Prism 6.00 software (Graphpad Software, La Jolla, CA, USA).

### 4.10. Wound Healing Assay and in Vitro Cell Invasion Assay

The wound healing assay was performed as described previously [7]. In brief, control and Rack1 knockdown cells were cultured to 100% confluence in six-well plates. Then, the cell monolayer was scratched using a 10-μL pipette tip to produce a straight wound. Cell debris was removed by washing the cells with medium. Afterward, the cells were incubated in culture medium containing 2% FBS at 37 °C for 0, 4, 8, 12, 24 and 36 h. At each time point, cell migration distance was measured under an inverted microscope. For the in vitro cell invasion assay, 200 μL of cell suspension (approximately 8 × 10^4^ cells) in serum-free medium were added into the Matrigel-coated Transwell insert (8-μm pore), and the lower insert was loaded with 10% FBS-containing medium. After incubation for 36 h at 37 °C, the cells invaded through the membrane were fixed and stained using the Three-Step Stain Set kit. The number of cells was counted under an inverted microscope. The assay was performed in triplicate and was repeated thrice.

### 4.11. Rh123 Efflux Assay

The Rh123 efflux assay was performed as described previously [7]. In brief, control and Rack1 knockdown cells were harvested with trypsinization, washed three times with PBS and adjusted to a density of 5 × 10^5^ cells/mL. The dye was added into the cells at a final concentration of 2 µg/mL. After incubation at 37 °C for 30 min, the cells were washed with PBS, resuspended in 500 µL of PBS and incubated for 15 min. The cells were further washed twice with PBS and immediately analyzed by flow cytometry (BD) using an excitation wavelength of 488 nm and an emission wavelength of 530 nm. The assay was performed in triplicate and repeated thrice.

### 4.12. Statistical Analysis

Data are presented as the mean ± SD. Differences between groups were analyzed by one-way ANOVA using the GraphPad Prism 6.00 software. *p*-values less than 0.05 (two-tailed) were considered statistically significant. An asterisk indicates that the *p*-value is less than 0.05.

## 5. Conclusions

In conclusion, our study identified Rack1 as a novel binding protein of P-gp. Rack1 functioned as a scaffold protein that facilitated the interaction of P-gp with Anxa2 and Src. Knockdown of Rack1 significantly inhibited the proliferation and invasiveness in drug-resistant cells. In addition, our finding showed that Rack1 regulated P-gp activity. We provided evidence that Rack1 is required for the integrity of this protein complex and is necessary for adriamycin-induced P-gp-mediated Anxa2 tyrosine phosphorylation and Erk1/2 activation. Thus, the findings in the present study add novel insights to the understanding of the mechanism employed by P-gp to promote the migration and invasion of MDR cancer cells.

## Supplementary Materials

Supplementary materials can be found at www.mdpi.com/1422-0067/17/10/1718/s1.
